# Using a novel data resource to explore heart rate during mountain and road running

**DOI:** 10.14814/phy2.13256

**Published:** 2017-04-19

**Authors:** Andrew Best, Barry Braun

**Affiliations:** ^1^ Department of AnthropologyUniversity of MassachusettsAmherstMassachusetts; ^2^ Department of Health and Exercise ScienceColorado State UniversityFort CollinsColorado

**Keywords:** Altitude, cardiac drift, hypoxia, Strava

## Abstract

Online, accessible performance and heart rate data from running competitions are posted publicly or semi‐publicly to social media. We tested the efficacy of one such data resource‐ Strava‐ as a tool in exercise physiology investigations by exploring heart rate differences in mountain racing and road racing running events. Heart rate and GPS pace data were gathered from Strava activities posted by 111 males aged 21–49, from two mountain races (Mt. Washington Road Race and Pike's Peak Ascent) and two road race distances (half marathon and marathon). Variables of interest included race finish time, average heart rate, time to complete the first half (by distance) of the race, time to complete the second half, average heart rate for both the first and second half, estimated maximal heart rate, and competitiveness (finish time as percentage of winning time). Mountain runners on average showed no change in heart rate in the second versus first half of the event, while road racers at the half marathon and marathon distances showed increased second‐half heart rate. Mountain runners slowed considerably more in the second half than road runners. Heart rate increases in road races were likely reflective of cardiac drift. Altitude and other demands specific to mountain racing may explain why this was not observed in mountain races. Strava presents enormous untapped opportunity for exercise physiology research, enabling initial inquiry into physiological questions that may then be followed by targeted laboratory studies.

## Introduction

Heart rate and GPS recording devices have become a common training tool for endurance athletes. Thousands post running and cycling activities on social media services such as Strava (www.strava.com), Movescount (www.movescount.com), and Training Peaks (www.trainingpeaks.com). Of these, Strava has the largest cache of public data (registration not required) and semipublic data (free registration required). Strava enables access to in situ data from thousands of athletic competitions that would require significant time and effort to collect through traditional research approaches. Standard data posted in Strava activities include pace and elevation, and sometimes heart rate and age. Some limitations are inherent: potentially relevant information, such as body mass, aerobic capacity, and training history are inaccessible without direct communication with individual athletes, which violates Strava's terms of use. Despite these constraints Strava represents an untapped “big‐data” source for exercise physiology research. Here, we explore the efficacy of this novel investigative approach through a comparative study of heart rate profiles of road running and mountain running competitions, events for which sufficient data is available on Strava.

Mountain running may be broadly defined as running or run/hiking over mountainous terrain and differs from traditional road racing in grade, altitude and terrain. The Mt. Washington Road Race (Gorham, NH) and the Pike's Peak Ascent (Manitou Springs, CO) are analogous in duration (but not distance) to the half marathon and marathon, respectively, traditional distances for road running competitions. Differences in heart rate and performance data between these races, gathered from Strava activities, may provide clues to the physiological demands unique to these two disciplines. Thus, a secondary goal of this study is to infer physiological differences between mountain and road running from studying heart rate profiles during these events. If identified, effects of differential biomechanics, cardiovascular physiology, and/or hypoxia could be studied directly in more targeted experimental studies.

## Methods

Mountain races sampled include the Mt. Washington Road Race (MWRR, 2009–2016) and the Pike's Peak Ascent or the ascent split from the Pike's Peak Marathon (PP, 2012–2016), held the same weekend over the same course but with a return downhill run. Candidate road races were identified by searching Strava for races with large samples of posted data. Chosen races included the Hartford Half Marathon (2011–2015), the Philadelphia Half Marathon (2011–2015), the Boston Athletic Association Half Marathon (2015), and the New York City Marathon (2015). It was necessary to sample from several half marathons over multiple years to achieve a sufficient sample size, and each of these races was chosen because these race courses are not excessively hilly nor is the second half of the race substantially more difficult than the first half. For any race, data were excluded for years where the ambient temperature was excessively warm. Also excluded were Strava activities that showed unrealistic HR profiles, such as precipitous changes, long periods of total stasis, or rapid fluctuations, all of which suggest heart rate monitor malfunction.

Most data were accessed through fully public or semipublic Strava pages (those requiring free site membership), but several runners submitted GPX data files directly after responding to Facebook requests and completing an informed consent document. Per Strava's request, Strava users were not contacted. Data were anonymized and participants were assigned study subject ID's. Heart rate and GPS data from 111 males aged 21–49 (mean 34.5 ± 6.4) were included in this study (see Table 1). Age, race finish time, winning time, and status of altitude residence (defined here as living at 3500' or higher) were determined by accessing race results posted on race websites. Variables of interest included: race finish time, age and residence (obtained from official race results); average heart rate (HR) throughout the race; time to complete the first half (by distance) of the race; time to complete the second half; and average HR for both the first and second half. HR_max_ was estimated using the Tanaka et al. (2001) equation for endurance trained men, 205—(0.6 *x* age). From these data other measures were calculated, including percentage of winning time (a measure of competitiveness), time difference to complete the first versus second half, heart rate difference in beats per minute (bpm) for the first versus second half, heart rate difference as a percentage of HR_max_ for the first versus second half, and overall heart rate as a percentage of HR_max._ Data were analyzed using SPSS statistical software v. 22 (IBM). *T*‐tests were performed for duration‐matched races and ANOVAs were used to examine differences between all test groups. Linear regressions were used to explore relationships between variables of interest. Finally, three additional MWRR competitors for whom heart rate data were not available were included to create a subgroup of four altitude‐acclimatized MWRR runners. All study protocol were reviewed and endorsed by the University of Massachusetts Human Subjects Review Board.

**Table 1:** Characteristics of participants in each race, mean ± SD

	All	MWRR	½ Marathon	Pikes Peak	Marathon	MWRR altitude subgroup
N	111	19	21	21	50	4
Age (years)	34.5 ± 6.4	35.0 ± 5.4	32.9 ± 6.7	37.0 ± 7.5	33.9 ± 5.9	31.8 ± 5.2
Race duration (hr:min:sec)	–	1:23:45 ± 0:08:20	1:25:42 ± 0:04:52	2:59:33 ± 0:11:47	2:54:43 ± 0:07:20	1:05:31 ± 0:10:07^c^
% of winning time	136.1 ± 9.5	142.8 ± 13.8^a^^,^ b	138.2 ± 10.1	133.1 ± 8.8^a^	133.9 ± 5.6^b^	111.3 ± 16.6^c^

a Significant difference (*P* < 0.01) between MWRR and Pikes Peak.

b Significant difference (*P* < 0.01) between MWRR and the Marathon. Runners in the shorter races, considered together, were slightly less competitive than those in longer races (*P* < 0.01). MWRR and the ½ marathon were significantly shorter in duration than Pikes Peak and the marathon (*P* < 0.0001).

c MWRR altitude subgroup had significantly lower % of winning time and race duration than all other groups (*P* < 0.01).

There were no significant differences in age between test groups nor significant differences in duration for matched races‐ MWRR (1:23:45 ± 0:08:20) versus the half marathon (1:25:42 ± 0:04:52), and PP (2:59:33 ± 0:11:47) versus the marathon (2:54:43 ± 0:07:20). Only one MWRR participant of 19 lived at altitude (in the initial sample) while only three of 21 PP runners did not. Runners' finish times ranged from 112% to 174% of the respective race winning time (mean 136 ± 9.5) and this measure was not different between duration and matched races. MWRR runners, however, were less competitive by this measure than PP and marathon runners (*P* < 0.01) and sampled runners in the shorter races considered together (MWRR and the ½ marathon) were slightly less competitive than the longer races (PP and the marathon; *P* < 0.01). Still, overall the runners in this study can be described as recreationally competitive. For example, most of the marathoners finished in under 3 h, a common benchmark of competitiveness, and the fastest ran 2 h 34 min, a performance that would earn a top‐10 finish in most American marathons outside of the major city marathons (Boston, New York, and Chicago). The subgroup of four MWRR runners from altitude was markedly more competitive than the larger study groups: two were race winners and one finished in second place, giving this subgroup an average finish time relative to the winner of 111.3%, significantly faster than any test group (*P* < 0.01).

## Results

**Figure 1:**
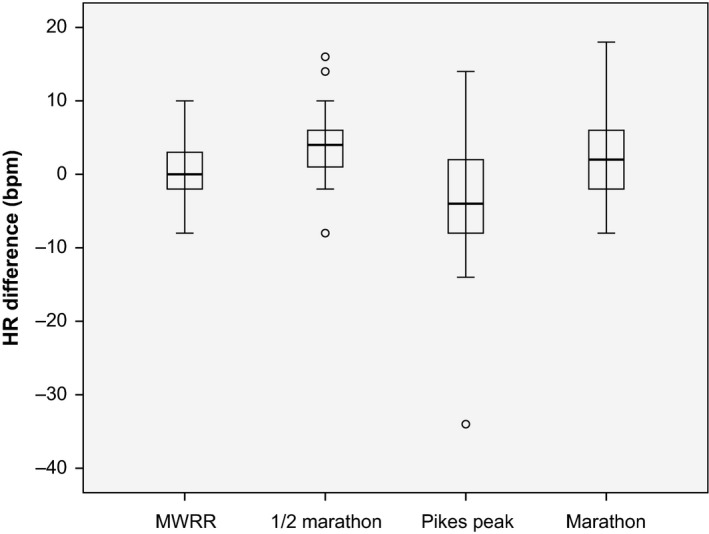
HR difference in the second versus first half of each race in beat per minute (bpm). Boxplots show first, second, and third quartiles, minimum and maximum, and outliers.

**Figure 2:**
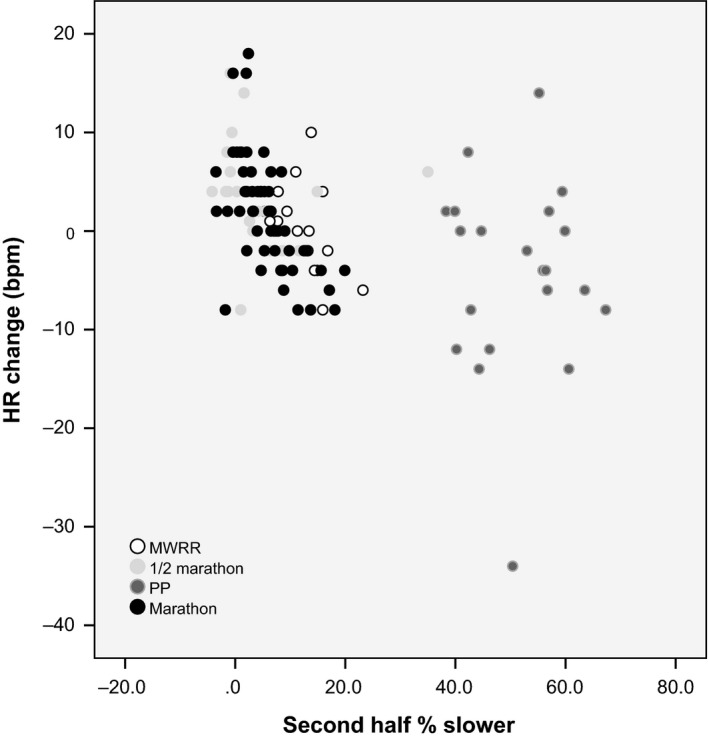
Relationship between slowing in the second half and HR change in the second half. Significant negative correlation for MWRR (*r* ^2^=0.224, *P* < 0.05) and the marathon (*r* ^2^=0.370, *P* < 0.0001).

Heart rate increased over the second half of both road events: half marathoners saw a 3.7 bpm increase (*P* < 0.01) and marathoners a 1.8 bpm increase (*P* < 0.05). There was no change in heart rate in the second versus first half of MWRR and HR decreased by 4.4 bpm at PP, though not quite significantly (*P* = 0.056; see Table 2 and Fig. 1). These differences are also reflected in percentage estimated HR_max_. Heart rate change was highly variable in the PP sample (SD=9.9 bpm) and there was a dramatic outlier whose HR dropped 34 bpm over the second half while slowing about the same as the average PP runner (see Fig. 2). When both mountain races were compared against both road races, the former were found to have significantly greater slowdown in the second half (*P* < 0.001) and a significantly different second half HR change (*P* < 0.01; see Table 3). This was true in duration‐matched pair comparisons as well: MWRR had greater second‐half slowing and less of a second‐half HR increase than the ½ marathon (*P* < 0.05), while PP had greater second‐half slowing and a drop in HR over the second half, as compared with the marathon where HR increased (*P* < 0.01). The four acclimatized runners comprising the MWRR altitude subgroup had similar second half slowing to other MWRR runners (15.4% ± 1.0 vs. 11.5% ± 4.8), significantly more than half marathoners and marathoners (*P* < 0.05) and significantly less than PP runners (*P* < 0.001). Runners in the shorter races (MWRR and the half marathon) displayed higher overall HR (bpm and as percentage estimated HR_max_; *P* < 0.05) and less second‐half slowing (*P* < 0.01).

**Table 2:** HR and pace results, mean ± SD

	MWRR	½ Marathon	Pikes Peak	Marathon	MWRR altitude subgroup
HR (bpm)	168.9 ± 8.3	171.1 ± 7.7	164.1 ± 9.8	166.5 ± 9.3	–
HR % estimated max	91.8 ± 4.7	92.4 ± 4.2	89.2 ± 4.7	90.2 ± 5.1	–
Second half % slower	11.5 ± 4.8^a^	4.3 ± 8.4	51.2 ± 8.8^b^	5.7 ± 5.4	15.4 ± 1.0^c^
HR change bpm	0.4 ± 4.3	3.7 ± 5.4^d^	−4.4 ± 9.9^e^	1.8 ± 6.1^d^	–
Change % est. HR_max_	0.2 ± 2.3	2.0 ± 2.9^d^	−2.4 ± 5.4^e^	1.0 ± 3.3	–

a MWRR slowed more than ½ marathoners (*P* < 0.01) and marathoners (*P* = 0.01).

b PP runners slowed more than all other groups (*P* < 0.0001).

c MWRR altitude runners slowed more than ½ marathoners and marathoners (*P* < 0.05).

d ½ Marathoners' (*P* < 0.01) and marathoners' (*P* < 0.05) HR increased in the 2^nd^ half.

e PP runners' HR decrease was significantly different from the marathoners' (*P* < 0.01) and ½ marathoners' (*P* = 0.001) HR increase.

**Table 3:** HR and pace for mountain versus road races and shorter versus longer races

	Mountain races	Road races	Shorter races	Longer races
HR (bpm)	166.4 ± 9.3	167.9 ± 9.0	170.0 ± 8.0^c^	165.8 ± 9.4
HR % estimated max	90.5 ± 4.8	90.9 ± 4.9	92.1 ± 4.4^c^	90.0 ± 4.9
Second half % slower	32.4 ± 21.3^a^	5.2 ± 6.4	7.7 ± 7.8^c^	19.1 ± 21.9
HR change bpm	−2.1 ± 8.0^b^	2.4 ± 5.9	2.1 ± 5.1	0.0 ± 7.9
Change % est. HR_max_	−1.1 ± 4.4^b^	1.3 ± 3.2	1.2 ± 2.8	0.0 ± 4.3

a Mountain races had greater second half slowing than road races (*P* < 0.0001).

b HR change in bpm and as % est. HR_max_ was significantly different in mountain versus road races (*P* < 0.001).

c Shorter races were characterized by higher HR (bpm and % est. HR_max_; *P* < 0.05) and less second half slowing (*P* < 0.01) than longer races.

Age was positively, though very weakly, correlated with slowing in the second half when all races were analyzed together (*r* ^2^=0.054; *P* < 0.05). No significant relationship was found in individual races. Percentage of winning time was positively and weakly correlated with HR for the marathon (*r* ^2^=0.085; *P* < 0.05) and inversely correlated with HR change in the second half of MWRR, both in bpm and percent estimated HR_max_ (*r* ^2^=0.207; *P* = 0.05); that is, less competitive runners had a smaller HR increase or larger decrease in the second half at these races. Considered all together, runners who slowed more in the second half of their race had less of a HR increase or a greater decrease both in bpm and percent estimated HR_max_ (*r* ^2^=0.187 and 0.189, respectively; *P* < 0.001). This relationship was significant only for MWRR runners (*r* ^2^=0.222 and 0.221, *P* < 0.05) and marathon runners (*r* ^2^=0.371 and 0.373, *P* < 0.001); there was almost no correlation at PP or the half marathon.

## Discussion

Our primary objective was to pilot the use of Strava's publicly and semi‐publicly available data in an exercise physiology investigation. The primary strength of this approach is that large quantities of data are available from a variety of competitions and training activities, enabling initial inquiry into questions that would otherwise be difficult to test. Questions dealing with relative performance will likely be more amenable to this approach than investigations of the underlying physiology as performance data are abundant but physiological data from Strava activities are limited. Indeed, heart rate is the only physiological measurement available; without knowledge of each subject's maximal heart rate, VO_2_‐max, blood lactate values and oxygen consumption during the event, physiological differences can only be inferred. Predictive equations based on age are routinely used to estimate HR_max_, but even the best of these (specific to endurance‐trained subjects and as used in this study) explains only 53% of the variation in HR_max_ between subjects (Tanaka et al. 2001). We could not control for effects of training history and runners unaccustomed to the specific demands of prolonged uphill running will surely respond and perform differently than well‐prepared competitors, a point that will be discussed further.

There are several ways in which Strava's utility to a researcher could be improved. First, recruiting users to record and submit heart rate and GPS data specifically for study purposes, which is currently against Strava's terms of use, could increase sample size and permit collection of additional information, including training characteristics. However, this would require extensive recruitment, obviating the primary strength of the approach piloted here (easy data access). Second, Strava or other athletic social media services may choose to incorporate a running power feature, allowing uploading of data from running power meters such as Stryd (www.stryd.com), a relatively new device which estimates power in watts; if accuracy is validated this could be a useful measure. Other services dedicated to tracking and analyzing athlete data—such as Movescount or Training Peaks, mentioned previously‐—may provide additional metrics, but at present these services do not host a public or semipublic data cache as large as Strava's. Finally, potential physiological differences identified through this approach could be explored further with targeted laboratory‐based studies where variables such as training history, climate, and terrain could be controlled and direct physiological measurements could be collected.

This study is also, to our knowledge, the first to demonstrate heart rate differences between mountain running and road running events. Compared to duration‐matched road races, participants in mountain races experienced no increase (MWRR) or a reduction (PP) in HR (both absolute and as percentage estimated HR_max_) and slowing pace over the second half of the race. The observed increases in HR in the second versus first half of the half marathon and marathon are not surprising: cardiac drift, an increase in HR without a concomitant increase in work output (i.e., running speed), has been demonstrated in one‐hour (Ekelund 1967; Mognoni et al. 1990) and 4‐hour (Dawson et al. 2005) exercise tests and is likely resultant from lower stroke volume concomitant with reduced plasma volume (Hamilton et al. 1991) and lower diastolic function (Dawson et al. 2005). There is little reason to speculate that these factors do not affect runners at MWRR and PP— races of similar duration to the half marathon and marathon, respectively— so maintenance of heart rate in those events must be attributable to a factor sufficiently robust that it masks the effect of drift. Three potential explanatory factors, not mutually exclusive, warrant further consideration.

### Altitude

The PP Ascent begins at 2382 m (7814 feet) and finishes at 4302 m (14 115 feet) while the Mt. Washington Road Race starts at 465 m (1526 feet) and finishes at 1917 m (6289 feet). The marathon and half marathons included in this study are held near sea level. Performance and cardiac function are undoubtedly impacted by the hypoxia encountered throughout the PP race: acute altitude exposure at simulated 4000 m (13 123 feet) increases heart rate and cardiac output during submaximal exercise to compensate for reduced arterial partial pressure of oxygen (PaO_2_), but maximal HR is reduced slightly, possibly due to reduced oxygen delivery to cardiac tissue (Stenberg et al. 1966) or increased production of epinephrine, which may have additional performance effects as it speeds uptake of glucose into muscle cells (Richardson et al. 1998). Wehrlin and Hallen (2006) found a 1.9 bpm decrease in maximal HR per 1000 m–in keeping with Stenberg et al.'s (1966) result at 4000 m– beginning at least as low as 1000 m, encompassing most of the altitudes encountered at MWRR. Thus, perhaps the lack of HR increase observed in mountain races does not reflect a reduction in percent HR_max_ (reduced aerobic effort), but rather lower HR_max_ resultant from hypoxia opposes cardiac drift and attenuates (MWRR) or completely negates (PP) a rise in heart rate in the second half of the race. Importantly, most participants in the PP Ascent live at and are ostensibly acclimatized to altitude, so many of their physiological responses during the race are not directly comparable with nonacclimatized runners; however, some HR effects persist even after acclimatization (Vogel et al. 1967).

Acclimatization also affords these runners a buffer against altitude‐induced performance decrements and thus a performance advantage relative to sea‐level runners (Mahe et al. 1974; Fulco et al. 2000). Most participants in the Mt. Washington Road Race are sea‐level residents and so any hypoxia experienced during the event is novel and acute. Little work has been done to evaluate HR responses to altitudes below 4000 m, but Wehrlin and Hallen's results (2006) suggest that Mt. Washington's altitude is sufficient to decrease maximal HR, as has been observed at altitudes equivalent to PP. Regardless, Mt. Washington's elevation should certainly incur a performance penalty, especially for non‐acclimatized runners. Reductions in VO_2_‐max have been observed relative to sea level values beginning at low altitudes: 580 m (Gore et al. 1996, 1997) and even right from sea level (Wehrlin and Hallen 2006). Thus, VO_2_‐max decreases linearly up to 3000 m, an impairment that appears to be more severe for endurance‐trained individuals (Lawler et al. 1988; Koistinen et al. 1995). This effect is not resultant from reduced maximal exercise intensity achieved in altitude tests: Wehrlin and Hallen (2006) found that performance, measured as time to exhaustion in running tests at simulated altitudes with speed kept constant, followed an observed 6% VO_2_‐max decrease per 1000 m. As the second half of MWRR ascends to an altitude 730 meters higher than the first half, we may predict a 4.3% VO_2_‐max penalty imposed by altitude in the second half, explaining part of the observed 11.5% slowing.

It is also possible that increasing altitude throughout both mountain races led to a slowing of pace while effort remained relatively unchanged. Enormous slowing at PP (51.2% ± 8.8) may be explained in part by the more extreme altitude and the increasing difficulty of the footing on the racecourse. However, as slowing was not associated with a decrease in HR for PP (*r* ^2^=0.001), increasing technical difficulty alone is unlikely to explain the vast discrepancy between PP slowing and marathon slowing (5.7%). MWRR is run on pavement, eliminating terrain as a complicating variable, so a direct comparison with the half marathon is easier‐ and indeed MWRR runners slowed far more than half marathoners (11.5% vs. 4.3%). Slowing pace at MWRR explains only 22% of the variability in HR decrease, so other factors must be operative.

### Pacing and psychological factors

An alternative or additional explanation for lack of HR increase together with slowing pace during mountain races is that runners reduce their effort as the race progresses due to psychological factors, or perhaps runners are not as adept at pacing themselves evenly in these events. This interpretation is supported by a weak but significant correlation between second‐half slowing and HR change at MWRR (*r* ^2^=0.202; *P* < 0.001): runners who slowed more had a greater decrease, or smaller increase, in HR compared with those who slowed less. However, 80% of the variation in HR change is not explained by slowing pace, and there is almost no correlation at all between these variables for PP (*r* ^2^=0.001). Additionally, the four altitude‐acclimatized MWRR athletes‐ three of whom are world class mountain runners (one is a world champion)‐ slowed just as much as their fellow MWRR competitors. This may suggest that altitude is not responsible for the observed slowing (and perhaps HR) effects as altitude acclimatization afforded no buffering against second‐half slowing. Alternatively, the fact that highly trained and experienced mountain runners slowed just as much as everyone else may suggest that pacing and psychological factors alone cannot account for slowing and HR changes, as these runners should be expected to be expertly prepared for the physical and psychological demands of mountain racing. Also, as acclimatization simply mitigates but does not eliminate altitude‐incurred diminishments in aerobic capacity, and VO_2_‐max declines linearly beginning from sea level, we may not expect acclimatized runners to slow less over the second half but rather to simply experience less of a performance declination overall relative to un‐acclimatized athletes.

### Muscle recruitment and biomechanical factors

The road races included in this study climb and descend no more than several hundred feet in total, while MWRR ascends about 4600' at an average grade of 12% and the PP Ascent climbs about 7800', also averaging 12% in grade. The biomechanics of uphill running differ significantly from level running: less eccentric work is performed by muscles and tendons, and none above 15% grade (Minetti et al. 1994), contributing to a higher energy cost. At grades steeper than 15% (which are briefly encountered at MWRR and PP) slopes of cost of transport for walking and running converge (Minetti et al. 1994), and above 28% grade, walking is more efficient than running (Giovanelli et al. 2016). Of course, in a race, efficiency is second to the primary goal of covering the course as quickly as possible. Many participants in mountain racing events, especially the slower racers, employ a mix of running and walking. So different are uphill biomechanics that the traditional definition of running gait may need to be modified to encompass locomotion lacking a true flight phase, but characterized by a bouncing gait instead of the inverted pendulum motion of walking. Such a gait has been described as “Groucho running” in a study of bent‐knee running on a level treadmill (McMahon et al. 1987) and “grounded running” in a study of ostriches (Rubenson et al. 2004), but these terms aptly describe the slower ranges of uphill human running (Giovanelli et al. 2016).

How do the incline‐specific biomechanics encountered in mountain racing affect physiology? Balducci et al. (2016) found that ten elite French mountain runners each achieved the same VO_2_‐max, blood lactate concentrations and heart rate in maximal tests on level ground, 12.5% slope, and 25% slope. Further, incline running performance was poorly predicted by level running performance, and there was significant inter‐individual variation in energy cost increase from level to uphill running: moving from 0% to 12.5% incline increased energy cost 50% for some subjects and 104% for others. These results inform the present study by suggesting that, in uphill‐trained subjects, (1) uphill racing absent altitude effects should not be characterized by different heart rate profiles and aerobic capacities; and (2) uphill running imposes unique challenges and specific training may have a strong effect on performance. However, this was a short test (<16 min) and the physiology of 1‐ to 3‐h mountain racing may be different; and importantly, the two mountain races we sampled do present altitude challenges. The observation that even highly trained mountain runners showed tremendous variation in uphill energy cost suggests that this effect may be even stronger in many of the athletes included in this study. A runner unaccustomed to the muscle recruitment patterns specific to mountain racing may prematurely exhaust particular muscle groups resulting in reduced performance relative to what he or she can achieve in a traditional road race (for which they are ostensibly better trained). For example, uphill running activates the vastus muscle group and the soleus to a greater extent than level running while other muscle groups are activated less (Gostill et al. 1974; Sloniger et al. 1997). It is possible that a runner unaccustomed to the demands of uphill running may exhaust the vastus and soleus muscles prematurely and be forced to reduce pace and aerobic effort as the race progresses. However, substantial second‐half slowing observed in this study's elite and altitude‐acclimatized subgroup—three athletes trained for the specific demands of mountain running—suggests that this effect may be minimal. A larger sample of elite runners could clarify this point.

Heart rate change in the second half of the marathon is more strongly correlated (*r* ^2^=0.370) with second‐half slowing than at MWRR (again, this correlation was inconsequential for the half marathon and PP). Here, perhaps is a good candidate application for the hypothesis of reduced HR due to reduced aerobic output. The eccentric muscle damage and diminishing glycogen stores incurred during a marathon may cause a slowing that is concurrent with, and causative of, a reduction in aerobic output. A moderate and significant correlation between slowing pace and HR decrease supports this explanation. Glycogen depletion should also be expected to pose a challenge for PP runners, but as HR change was not associated with changing pace, glycogen depletion can only minimally explain HR and pace changes.

Other observed significant correlations are most likely specious. Less competitive MWRR runners displayed a greater reduction or smaller increase in HR over the second half, but competitiveness was not correlated with second‐half slowing at this or any race. The very weak correlation between age and second‐half slowing disappeared when examined for individual races. Less competitive marathoners had higher HR, but an *r*‐squared value of 0.085 and a lack of correlation between competitiveness and any other variable suggest this is a false positive. Finally, the higher HR (as bpm and percentage estimated HR_max_) in the shorter‐duration events confirms long‐established observations that relative intensity is inversely related to exercise duration.

In summary, uphill mountain racing does not appear to be characterized by the continually increasing heart rate seen in the half marathon and marathon. Our three hypotheses for this phenomenon could be investigated with a laboratory based study with subjects completing race effort runs on flat and uphill grades. This would control for terrain, altitude, and variability in interindividual responses (each subject could complete both flat and uphill race efforts), and would permit collection of physiological data (VO_2_, RER, blood lactate, etc.) and training history.

## Conclusions

Strava's performance and heart rate data are a useful and novel resource for exercise science investigations provided that research questions are carefully articulated in consideration of the strengths and limitations of this approach. Competitors in mountain races slowed more than their counterparts in duration‐matched road races. Mountain racing is characterized by a maintained or decreased heart rate in the second versus first half of the event, while road racing at the half marathon and marathon distances is characterized by an increasing heart rate. It is unclear whether or how altitude or demands specific to uphill running explain this difference. This study demonstrates how Strava data can be used in an inquiry into a physiological or performance question; results may then be used to inform a targeted laboratory‐based study.

## Conflict of Interest

The authors declare no conflicts of interest.

## References

[phy213256-bib-0001] Balducci, P. , M. Clémençon , B. Morel , G. Quiniou , D. Saboul , and C. A. Hautier . 2016 Comparison of level and graded treadmill tests to evaluate endurance mountain runners. J. Sports Sci. Med. 15:239–246. 27274660 PMC4879436

[phy213256-bib-0002] Dawson, E. A. , R. Shave , K. George , G. Whyte , D. Ball , D. Gaze , et al. 2005 Cardiac drift during prolonged exercise with echocardiographic evidence of reduced diastolic function of the heart. Eur. J. Appl. Physiol. 94:305–309. 15765237 10.1007/s00421-005-1318-3

[phy213256-bib-0003] Ekelund, L. G. 1967 Circulatory and respiratory adaptation during prolonged exercise. Acta Physiol. Scand. Suppl. 292:1. 4168491

[phy213256-bib-0004] Fulco, C. S. , P. Rock , and A. Cymerman . 2000 Improving athletic performance: is altitude residence or altitude training helpful? Aviat. Space Environ. Med. 71:162–171. 10685591

[phy213256-bib-0005] Giovanelli, N. , A. L. R. Ortiz , K. Henninger , and R. Kram . 2016 Energetics of vertical kilometer foot races; is steeper cheaper? J. Appl. Physiol. 120:370–375. 26607247 10.1152/japplphysiol.00546.2015

[phy213256-bib-0006] Gore, C. J. , A. G. Hahn , G. C. Scroop , D. B. Watson , K. I. Norton , R. J. Wood , et al. 1996 Increased arterial desaturation in trained cyclists during maximal exercise at 580 m altitude. J. Appl. Physiol. 80:2204–2210. 8806931 10.1152/jappl.1996.80.6.2204

[phy213256-bib-0007] Gore, C. J. , S. C. Little , A. G. Hahn , G. C. Scroop , K. I. Norton , P. C. Bourdon , et al. 1997 Reduced performance of male and female athletes at 580 m altitude. Eur. J. Appl. Physiol. Occup. Physiol. 75:136–143. 9118979 10.1007/s004210050138

[phy213256-bib-0008] Gostill, D. L. , E. Jansson , P. D. Gollnick , and B. Saltin . 1974 Glycogen utilization in leg muscles of men during level and uphill running. Acta Physiol. Scand. 91:475–481. 4432759 10.1111/j.1748-1716.1974.tb05703.x

[phy213256-bib-0009] Hamilton, M. T. , J. Gonzalez‐Alonso , S. J. Montain , and E. F. Coyle . 1991 Fluid replacement and glucose infusion during exercise prevent cardiovascular drift. J. Appl. Physiol. 71:871–877. 1757323 10.1152/jappl.1991.71.3.871

[phy213256-bib-0010] Koistinen, P. , T. Takala , V. Martikkala , and J. Leppäluoto . 1995 Aerobic fitness influences the response of maximal oxygen uptake and lactate threshold in acute hypobaric hypoxia. Int. J. Sports Med. 16:78–81. 7751080 10.1055/s-2007-972968

[phy213256-bib-0011] Lawler, J. , S. K. Powers , and D. Thompson . 1988 Linear relationship between VO_2_‐max and VO_2_‐max decrement during exposure to acute hypoxia. J. Appl. Physiol. 64:1486–1492. 3378983 10.1152/jappl.1988.64.4.1486

[phy213256-bib-0012] Mahe, J. T. , L. G. Jones , and L. H. Hartley . 1974 Effects of high‐altitude exposure on submaximal endurance capacity of men. J. Appl. Physiol. 37:895–898. 4436221 10.1152/jappl.1974.37.6.895

[phy213256-bib-0013] McMahon, T. A. , G. Valian , and E. C. Frederick . 1987 Groucho running. J. Appl. Physiol. 62:2326–2337. 3610929 10.1152/jappl.1987.62.6.2326

[phy213256-bib-0014] Minetti, A. E. , L. P. Ardigo , and F. Saibene . 1994 Mechanical determinants of the minimum energy cost of gradient running in humans. J. Exp. Biol. 195:211–225. 7964412 10.1242/jeb.195.1.211

[phy213256-bib-0015] Mognoni, P. , M. D. Sirtori , F. Lorenzelli , and P. Cerretelli . 1990 Physiological responses during prolonged exercise at the power output corresponding to the blood lactate threshold. Eur. J. Appl. Physiol. Occup. Physiol. 60:239–243. 2357977 10.1007/BF00379389

[phy213256-bib-0016] Richardson, R. S. , E. A. Noyszewski , J. S. Leigh , and P. D. Wagner . 1998 Lactate efflux from exercising human skeletal muscle: role of intracellular. J. Appl. Physiol. 85:627–634. 9688741 10.1152/jappl.1998.85.2.627

[phy213256-bib-0017] Rubenson, J. , D. Heliams , D. G. Lloyd , and P. A. Fournier . 2004 Gait selection in the ostrich: mechanical and metabolic characteristics of walking and running with and without an aerial phase. Proc. R. Soc. Lond. B Biol. Sci. 271:1091–1099. 10.1098/rspb.2004.2702 PMC1691699 15293864

[phy213256-bib-0018] Sloniger, M. A. , K. J. Cureto , B. M. Prior , and E. M. Evans . 1997 Lower extremity muscle activation during horizontal and uphill running. J. Appl. Physiol. 83:2073–2079. 9390983 10.1152/jappl.1997.83.6.2073

[phy213256-bib-0019] Stenberg, J. , B. Ekblom , and R. Messin . 1966 Hemodynamic response to work at simulated altitude, 4000 m. J. Appl. Physiol. 21:1589–1594. 5923231 10.1152/jappl.1966.21.5.1589

[phy213256-bib-0020] Tanaka, H. , K. D. Monahan , and D. R. Seals . 2001 Age‐predicted maximal heart rate revisited. J. Am. Coll. Cardiol. 37:153–156. 11153730 10.1016/s0735-1097(00)01054-8

[phy213256-bib-0021] Vogel, J. A. , J. E. Hansen , and C. W. Harris . 1967 Cardiovascular responses in man during exhaustive work at sea level and high altitude. J. Appl. Physiol. 23:531–539. 6053679 10.1152/jappl.1967.23.4.531

[phy213256-bib-0022] Wehrlin, J. P. , and J. Hallen . 2006 Linear decrease in VO_2_‐max and performance with increasing altitude in endurance athletes. Eur. J. Appl. Physiol. 96:404–412. 16311764 10.1007/s00421-005-0081-9

